# Hearing Loss and Dementia: Risk Factor, Early Marker, or Both?

**DOI:** 10.3390/healthcare14121687

**Published:** 2026-06-12

**Authors:** Ljiljana Cvorovic, Ana Jotic, Bojana Bukurov, Saša Jakovljevic, Simona Aleksic, Katarina Jovanovic

**Affiliations:** 1 Faculty of Medicine, University of Belgrade, 11000 Beograd, Serbia; ana.jotic@med.bg.ac.rs (A.J.); bojana.bukurov@med.bg.ac.rs (B.B.); jakovljevicsasa81@gmail.com (S.J.); 2 Clinic for Otorhinolaryngology and Maxillofacial Surgery, University Clinical Centre Serbia, 11000 Belgrade, Serbia; simonarandjelovic@gmail.com (S.A.); jovanovickatarina323@gmail.com (K.J.)

**Keywords:** hearing loss, dementia, cognitive dysfunction, age-related hearing loss, presbycusis, auditory–cognitive interaction, neuroplasticity, auditory rehabilitation

## Abstract

**Background/Objectives:** Hearing loss and dementia are highly prevalent conditions in older adults and represent a growing public health challenge. Over the past decade, a substantial body of epidemiological evidence has demonstrated a consistent association between age-related hearing loss and cognitive dysfunction, including incident dementia. However, the nature of this relationship remains incompletely understood. **Methods:** This narrative review provides a structured overview of current evidence, focusing on epidemiological findings, mechanistic pathways, and clinical implications. Hearing loss has been associated with both accelerated cognitive decline and increased dementia risk, with a clear severity–impact relationship. **Results:** Several interacting mechanisms have been proposed, including increased cognitive load, structural and functional brain changes, social isolation, and shared vascular and metabolic risk factors. Emerging concepts such as the “auditory brain” and central auditory dysfunction further suggest that hearing impairment may also represent an early manifestation of neurodegenerative processes. Intervention studies have yielded mixed results. While hearing rehabilitation improves communication and quality of life, randomized evidence has not consistently demonstrated a reduction in cognitive decline in the general population, but potential benefits may exist in higher-risk subgroups. Increasing attention has been directed toward the role of neuroplasticity, with evidence suggesting that delayed intervention may limit the effectiveness of rehabilitation due to long-standing auditory deprivation. **Conclusions:** Taken together, current evidence suggests that hearing loss may represent both a potentially modifiable risk factor and an early marker of cognitive decline. Early identification and timely management of hearing impairment may therefore play an important role in maintaining cognitive and brain health and improving quality of life in older adults.

## 1. Introduction

Hearing loss and dementia are among the most prevalent chronic conditions affecting the aging population and represent a major and escalating public health challenge. Globally, more than 460 million people are affected by disabling hearing loss. At the same time, the number of individuals with dementia is projected to exceed 150 million by 2050, particularly in low- and middle-income countries [1]. Given their strong association with aging, these conditions frequently coexist, particularly in adults over 65 years of age, thereby amplifying their individual and societal burden.

Over the past decade, converging epidemiological evidence has consistently demonstrated a robust association between sensory impairment and cognitive decline in older adults, including incident dementia. In a seminal prospective analysis from the Baltimore Longitudinal Study of Aging, hearing loss was independently associated with incident dementia in a clear dose–response relationship between hearing loss severity and dementia risk, with risk increasing up to fivefold in individuals with severe impairment [2]. These findings have been replicated across multiple longitudinal cohorts and meta-analyses, confirming that hearing impairment is associated with accelerated cognitive decline and increased dementia risk [1,3,4,5,6,7].

This consistent association has led to the inclusion of hearing loss as a potentially modifiable risk factor for dementia in the Lancet Commission reports [4,8]. However, despite the growing body of epidemiological evidence, the causal nature of this relationship has yet to be definitively established. Most available data derive from observational studies, which are inherently susceptible to residual confounding and reverse causation. As highlighted in recent critical appraisals, hearing loss may represent a causal factor, an early marker of neurodegeneration, or a correlate of shared pathological processes, including vascular and metabolic dysfunction [9,10].

Multiple pathophysiological pathways have been proposed to explain this association, including increased cognitive load due to degraded auditory input, structural brain alterations such as accelerated cortical and hippocampal atrophy, reduced social engagement, and shared vascular and neurodegenerative risk factors [1,11,12]. More recently, a paradigm shift has emerged. Hearing is increasingly recognized as a cognitive function rather than a purely sensory process. The concept of the “auditory brain” emphasizes that auditory processing relies on distributed neural networks that are intrinsically vulnerable to neurodegenerative disease, challenging the traditional separation between peripheral and central hearing [13]. Within this framework, auditory dysfunction may represent an early manifestation of dementia rather than solely a modifiable risk factor.

The clinical implications of this relationship are substantial. If hearing loss contributes to cognitive decline, early identification and management could offer a scalable strategy for reducing dementia risk. Conversely, if hearing impairment reflects early neurodegenerative changes, it may serve as a useful marker for identifying individuals at increased risk. Current evidence on hearing interventions remains mixed, with randomized data showing limited overall cognitive benefit, although effects may be present in selected high-risk populations [9,14,15,16].

## 2. Materials and Methods

This narrative review provides a structured overview of the relationship between hearing loss, cognitive decline, and dementia, their underlying mechanisms, and clinical implications, with a particular focus on whether hearing loss represents a modifiable risk factor, an early marker, or both.

A literature search was performed between January and March 2026 using PubMed and Google Scholar databases. Publications from January 2000 to March 2026 were considered, with particular emphasis on studies published within the last decade. Search terms included combinations of “hearing loss”, “age-related hearing loss”, “presbycusis”, “hearing impairment”, “cognitive decline”, “cognitive dysfunction”, “dementia”, “Alzheimer disease”, “auditory brain”, “central auditory processing”, “hearing aids”, “cochlear implantation”, and “auditory rehabilitation”.

Priority was given to longitudinal cohort studies, systematic reviews, meta-analyses, randomized controlled trials, and landmark epidemiological investigations. Additional studies were identified through manual screening of the reference lists of relevant articles. Studies were selected based on their relevance to one or more of the following domains: (1) epidemiological associations between hearing loss and cognitive decline or dementia; (2) biological and neurocognitive mechanisms linking auditory and cognitive dysfunction; (3) effects of hearing rehabilitation interventions on cognitive outcomes; and (4) emerging concepts related to central auditory dysfunction and neurodegeneration.

Studies focused exclusively on pediatric populations, non-age-related hearing disorders, or conditions unrelated to cognitive outcomes were generally excluded unless they provided important mechanistic insights relevant to the topic.

Given the narrative nature of this review, formal systematic review procedures, quality scoring, and quantitative synthesis were not performed.

## 3. Epidemiological Evidence

Epidemiological evidence consistently demonstrates a relationship between hearing loss and risk for cognitive decline. This relationship shows a clear dose–response pattern. In the Baltimore Longitudinal Study of Aging, dementia risk increased by approximately 27% for every 10 dB increase in hearing thresholds, supporting a dose–response relationship rather than a simple threshold effect [2]. In addition, hearing loss has been associated with faster cognitive decline over time, with individuals showing steeper reductions in global cognition and processing speed [17]. Meta-analysis by Loughrey et al. [3] found that individuals with age-related hearing loss had 1.22-fold higher odds of cognitive impairment (95% CI: 1.09–1.36) and 1.28-fold higher odds of dementia (95% CI: 1.02–1.59) compared to those with normal hearing. Furthermore, longitudinal observations suggest that hearing decline may precede the onset of dementia, indicating that auditory dysfunction could appear early in the disease process (Table 1).

Hearing loss in epidemiological studies is most commonly assessed using pure-tone audiometry, which measures peripheral hearing sensitivity across key speech frequencies (typically 0.5–4 kHz). While this method provides a standardized measure of hearing thresholds, it primarily reflects sound threshold detection rather than real-world auditory processing. Central auditory function, including speech perception in noisy environments, depends on both sensory encoding and higher-level cognitive processing and may better reflect real-world communication [20,21,22]. In addition, self-reported hearing captures subjective and functional aspects of hearing ability that are not fully reflected by audiometric thresholds, further highlighting the multidimensional nature of hearing assessment [23].

Large cohort studies provide further insight into how this association develops. In the ARIC study, hearing loss was more strongly related to decline in executive functions and processing speed than to memory [5], suggesting involvement of frontal and subcortical brain networks. Similarly, the Caerphilly Prospective Study demonstrated that phonological processing demands, measured through performance on auditory verbal tasks requiring perception, encoding, and recall of spoken words, were more predictive of incident dementia than pure auditory thresholds alone [6]. This finding highlights the importance of central auditory–cognitive processes, rather than peripheral hearing alone. These observations are supported by broader sensory–cognitive aging frameworks, which demonstrate strong correlations between sensory and cognitive function across the lifespan, independent of chronological age [24]. Evidence from administrative health data further supports this association, showing that clinically diagnosed hearing impairment is linked to increased dementia incidence in large real-world populations [18]. More recent prospective data using formal behavioral audiometric testing have confirmed these findings, strengthening the evidence by reducing measurement bias [19].

Meta-analyses and pooled data confirm the overall association but also reveal important variability. Hearing loss is consistently associated with cognitive impairment and dementia across studies [3,7], but effect sizes vary by study design and methodology. One key limitation is how hearing and cognition are measured. Pure-tone audiometry may not reflect real-world listening abilities, especially in complex environments, while cognitive testing may be influenced by unrecognized hearing impairment, potentially leading to overestimation of cognitive decline [25]. In addition, socioeconomic factors play an important role. Hearing loss is more common in socially deprived populations, which are also at increased risk of cognitive decline, partly due to higher exposure to health risks and reduced access to care, making it difficult to disentangle independent effects [26].

Adjustment for shared risk factors, such as cardiovascular disease, diabetes, and social determinants, often reduces the strength of the association between hearing loss and dementia [9,10]. This suggests that hearing loss may partly reflect broader processes related to aging and general health. However, the association is not fully explained by these factors, indicating that hearing loss may still contribute independently.

An important limitation of the current literature is that age-related hearing loss is often treated as a homogeneous condition. However, presbycusis encompasses several pathological subtypes, including sensory, neural, metabolic (strial), mechanical, and mixed forms. These subtypes differ in their underlying cochlear pathology and may have distinct implications for cognitive outcomes. Most epidemiological studies rely on pure-tone audiometry and do not distinguish between presbycusis subtypes, making it difficult to determine whether specific biological mechanisms confer differential dementia risk.

An additional limitation of the current literature is that dementia is often treated as a single clinical entity. However, Alzheimer’s disease, vascular dementia, dementia with Lewy bodies, and mixed dementia differ substantially in their underlying pathology and may exhibit distinct relationships with auditory dysfunction. Alzheimer’s disease may preferentially affect central auditory networks involved in higher-order auditory processing, whereas vascular dementia shares important vascular risk factors with hearing loss. Dementia with Lewy bodies may present with prominent auditory perceptual disturbances and hallucinations [13]. Future studies should therefore consider dementia subtypes separately rather than assuming a uniform auditory–cognitive relationship across all neurodegenerative disorders.

## 4. Mechanisms and Interpretations Linking Hearing Loss and Cognitive Decline

Several mechanisms have been proposed to explain the relationship between hearing loss and cognitive decline. These mechanisms are not mutually exclusive and likely interact over time [9,10].

**Figure 1:**
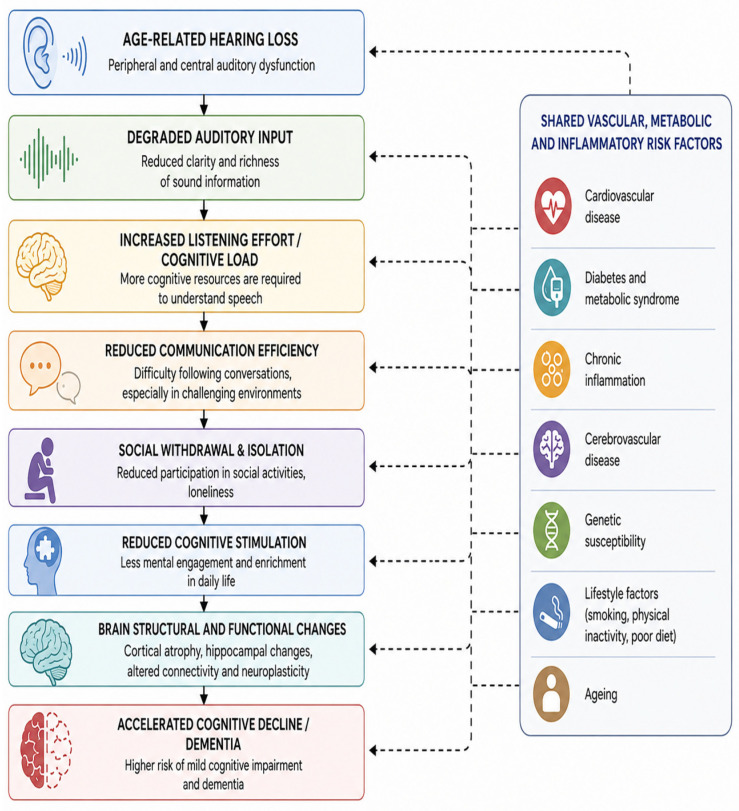
Proposed pathways linking age-related hearing loss and cognitive decline.

Rather than acting independently, these mechanisms are likely interconnected and may form a self-reinforcing pathway linking auditory dysfunction and cognitive decline. Degraded auditory input may initially increase listening effort and cognitive load, which can subsequently contribute to reduced social engagement, alterations in brain structure and function, and greater vulnerability to neurodegenerative processes. Shared vascular and metabolic risk factors may further amplify these effects. Consequently, hearing loss and dementia should be viewed within a multidimensional framework in which sensory, cognitive, behavioral, and biological mechanisms interact throughout the aging process (Figure 1).

One of the most widely discussed mechanisms is increased cognitive load. When auditory input is degraded, the brain must allocate more resources to speech decoding, leaving fewer available for memory and higher cognitive functions [10,11]. This process, often referred to as “listening effort,” may lead to sustained cognitive strain. The finding that phonological processing demands predict dementia risk better than hearing thresholds alone [6] further supports the idea that the cognitive burden of hearing, rather than hearing loss itself, plays a key role. Experimental and neuroimaging studies have shown that degraded auditory input requires increased allocation of cognitive resources, particularly affecting working memory and executive function, with compensatory recruitment of frontal brain regions [27,28,29].

Neuroimaging studies suggest that hearing loss is associated with structural and functional changes in the brain. Reduced auditory input has been associated with accelerated atrophy in the auditory cortex and related brain regions, including areas involved in memory and executive functions [11,13]. The concept of the “auditory brain” highlights that hearing is not a purely peripheral function but depends on widespread neural networks [13]. Structural neuroimaging studies have demonstrated reduced gray matter volume in the primary auditory cortex and temporal lobe regions, as well as altered white matter integrity in central auditory pathways. These changes have been observed in association with hearing loss and may reflect both sensory deprivation and underlying neurodegeneration. Evidence from imaging studies further suggests accelerated atrophy in temporal regions involved in auditory processing and memory in individuals with hearing loss [30,31,32].

Hearing loss can impair communication, leading to reduced social interaction and increased social isolation. Social isolation is a well-established risk factor for cognitive decline and dementia [4]. Individuals with untreated hearing loss may withdraw from conversations and social activities, resulting in decreased cognitive stimulation. Over time, reduced engagement may contribute to cognitive decline through decreased activation of cognitive networks. This pathway may also interact with other factors, such as depression and reduced quality of life, further amplifying the risk. Beyond behavioral effects, social isolation may contribute to cognitive decline through neurobiological mechanisms, including increased stress response, dysregulation of the hypothalamic–pituitary–adrenal axis, and systemic inflammation, all of which have been linked to neurodegenerative processes [33,34].

Hearing loss and dementia share several risk factors, including aging, vascular disease, diabetes, and inflammation [9,10,12]. These common pathways may contribute to both peripheral auditory dysfunction and central neurodegeneration. For example, vascular changes can affect both cochlear function and cerebral perfusion. Similarly, metabolic and inflammatory processes may contribute to damage in both auditory and cognitive systems.

A central question is whether hearing loss contributes causally to dementia or represents an early manifestation of neurodegenerative processes. Evidence supporting a causal role includes longitudinal studies demonstrating that hearing loss precedes cognitive decline, as well as a clear dose–response relationship across levels of severity [2,17]. Proposed mechanisms, such as increased cognitive load and reduced social engagement, provide biologically plausible pathways. However, hearing loss may also represent an early manifestation of brain changes. Within the framework of the “auditory brain,” auditory processing depends on distributed neural networks that are vulnerable to neurodegeneration [13]. This may help explain why central auditory dysfunction can occur early and may precede or accompany measurable peripheral hearing loss. A third explanation is that both conditions may arise from shared risk factors, including vascular and metabolic disease. The relative contribution of these mechanisms likely varies across individuals and disease stages. Rather than supporting a single explanatory framework, current evidence is consistent with causal, early-marker, and shared-pathology models, each with distinct strengths, limitations, and implications for clinical practice and future research (Table 2).

## 5. Hearing Interventions and Their Impact on Cognition

**Figure 2:**
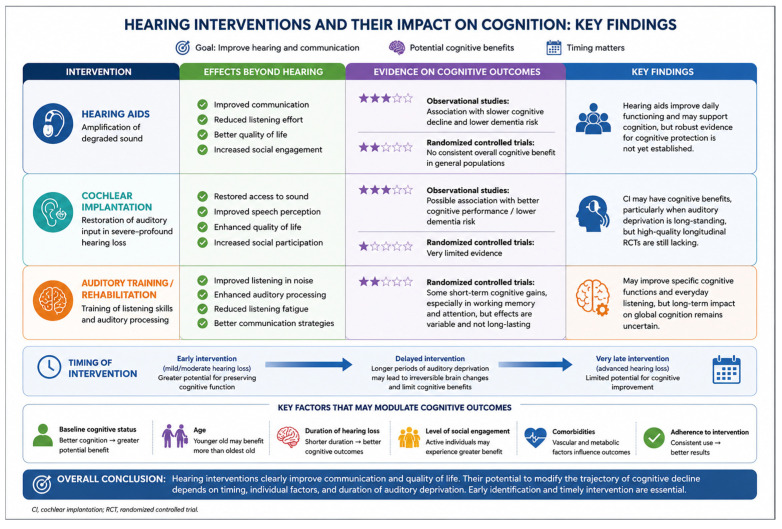
Summary of current evidence regarding hearing interventions and cognitive outcomes.

If hearing loss contributes to cognitive decline, then auditory rehabilitation could represent a practical strategy to slow this process. Hearing interventions clearly improve communication and quality of life, but their effect on cognition and dementia risk is still under investigation [9,14,35]. The current evidence regarding hearing interventions and their potential effects on cognitive outcomes is summarized in Figure 2.

### 5.1. Hearing Aids and the Impact on Cognition

Observational studies have suggested that hearing aid use may be associated with better cognitive outcomes. Some longitudinal studies have reported slower cognitive decline among hearing aid users compared with non-users [15], and meta-analytic data suggest a reduced risk of cognitive decline in treated individuals [35,36]. However, these findings are likely influenced by residual confounding and selection bias, as hearing aid users often differ from non-users in education, health status, and access to care [9].

The strongest evidence so far comes from the ACHIEVE trial, a multicenter randomized controlled trial that compared a hearing intervention with an active control group receiving health education in older adults with untreated hearing loss [16]. In the overall cohort, the intervention did not significantly reduce cognitive decline over three years. However, a prespecified subgroup analysis showed a slower rate of decline among participants at higher risk. These findings suggest that the cognitive effects of hearing rehabilitation may depend on baseline risk and population characteristics rather than being uniform across all individuals.

Several factors may explain the neutral overall findings of the ACHIEVE trial. First, the follow-up period of three years may have been insufficient to detect meaningful differences in long-term cognitive trajectories, given the typically slow progression of neurodegenerative disease. Second, participants in the primary prevention cohort were relatively healthy and may have been at lower risk for cognitive decline, reducing the likelihood of demonstrating a treatment effect. Third, hearing interventions may primarily improve functional cognitive performance through reduced listening effort, enhanced communication, and greater social engagement rather than directly modifying underlying neurodegenerative pathology. These considerations highlight the importance of patient selection, intervention timing, adherence to rehabilitation, and longer follow-up periods in future randomized studies. Importantly, hearing rehabilitation may provide cognitive benefit even without modifying the underlying neurodegenerative process. Improved auditory input reduces listening effort, enhances communication, and supports social engagement, which in turn may help maintain functional cognitive performance and delay the clinical expression of impairment [9,14]. This distinction is particularly relevant in individuals with mild cognitive impairment or early dementia, where interventions may improve daily functioning and quality of life despite ongoing neurodegeneration.

### 5.2. Cochlear Implantation and the Impact on Cognition

Cochlear implantation in adults with severe to profound hearing loss has been shown to confer several beneficial effects on cognition, quality of life, and mental health, with some evidence pointing towards its potential to delay cognitive decline [37,38]. Prospective studies following recipients for up to 24 months post-implantation have demonstrated significant improvement in various cognitive domains, including attention, working memory, inhibition, recall, and verbal fluency, with these cognitive gains being particularly notable in individuals who had lower cognitive reserve prior to implantation [39].

It was also shown that cochlear implant users experience improved executive function and working memory, alongside stability in attention, psychomotor function, and visual learning, compared to community-dwelling peers with untreated hearing loss who tended to show cognitive decline over time [40]. In a long-term observational study following profoundly deaf older adults (aged 65+) after cochlear implantation, a low rate of progression from mild cognitive impairment to dementia over nearly 7 years was found, with some individuals showing cognitive improvements post-implantation [41].

In addition to cognitive effects, cochlear implants improve health-related quality of life in elderly recipients, strengthening the communication ability, reducing feelings of loneliness, and improving emotional well-being post-implantation [42].

Although cochlear implantation in older adults with dementia or cognitive impairment can have beneficial outcomes, particularly in speech recognition and quality of life, cognitive improvements remain less certain and require further research. A recent review of 222 cochlear implant recipients with cognitive impairment or dementia found that these patients benefit in terms of improved speech recognition, although the degree of benefit may be somewhat less compared to cognitively healthy individuals [43]. It should be stressed that most cochlear implant studies among older adults exclude individuals with advanced dementia (i.e., selection bias), but the above findings indicate that implantation should not be categorically withheld from candidates with cognitive decline.

### 5.3. Timing of the Intervention

The timing of the intervention appears to be critical. Prolonged auditory deprivation is associated with structural and functional brain changes, including cross-modal reorganization, where auditory cortical areas are recruited for other sensory functions [44]. Although often interpreted as an adaptive response to sensory deprivation, the long-term functional consequences of cross-modal reorganization remain incompletely understood and may vary according to the underlying neurodegenerative context. The capacity for neural adaptation declines with longer durations of untreated hearing loss. Delayed intervention has been associated with reduced improvement in speech perception and higher-level auditory processing, even with appropriate amplification [45,46].

Auditory rehabilitation with hearing aids has been shown to induce beneficial neuroplastic changes, including improved cortical responses and partial reversal of cross-modal reorganization, particularly when intervention occurs early [42,43,44,45,46,47,48,49]. Earlier fitting of hearing aids, especially before dementia develops or in early stages of cognitive impairment, seems more likely to preserve cognition and delay decline [36,50]. Regardless, once significant dementia is established, hearing aids may be less effective at improving cognition but still provide benefits in reducing depressive symptoms, improving communication, and decreasing caregiver burden [51]. Cognitive status also influences the persistence of hearing aid use. Individuals with better baseline cognition are more likely to adhere to hearing aids, which in turn is associated with a lower risk of dementia [52].

In older adults with severe hearing loss, the timing of cochlear implantation plays an important role in neuroplasticity outcomes and is associated with cognitive recovery. Older adults implanted earlier in the trajectory of hearing loss may experience greater cognitive gains, especially those with lower cognitive function before surgery, suggesting a heightened neuroplastic response to auditory restoration when intervention is not delayed [53]. Similarly, adults over 70 demonstrated significant gains in working memory and processing speed 12 months after implantation, further emphasizing the capacity for neuroplastic cognitive improvement even in advanced age [54]. Cognitive benefits appear to plateau after about one to two years post-implantation, suggesting that the intervention should be performed before severe or irreversible neurodegeneration occurs [55].

This research highlights the interaction between neuroplasticity and neurodegeneration. Early in the course of hearing loss, neuroplastic mechanisms may support compensation through increased neural recruitment and reorganization. Over time, however, persistent sensory deprivation may contribute to structural brain changes that increase vulnerability to cognitive decline. Importantly, these changes do not necessarily represent neurodegenerative disease per se but may interact with underlying neurodegenerative processes [56].

Emerging evidence suggests that early neurodegenerative changes may affect central auditory pathways, further supporting the close interaction between auditory and cognitive aging processes [57].

## 6. Clinical and Public Health Implications

Hearing loss should be viewed as an integral component of health in older adults, not only because of its impact on communication but also due to its association with cognitive decline. Current evidence does not support the conclusion that treating hearing loss prevents dementia. However, it clearly supports early identification and management as part of comprehensive care.

From a clinical perspective, routine hearing assessment is important in older adults, particularly in those with cognitive complaints or increased risk of cognitive decline. Standard pure-tone audiometry remains essential, but it may not fully reflect real-world listening difficulties. Greater emphasis on speech understanding and functional hearing assessment may provide more clinically relevant information, especially in patients with suspected central auditory involvement, which often accompanies peripheral hearing loss and is linked with accelerated cognitive decline and dementia [58].

Hearing rehabilitation, including the use of hearing aids and cochlear implants, should be offered when indicated. Even if these interventions do not fully modify the underlying neurodegenerative process, they can reduce listening effort, improve communication, and support social participation. These effects may help maintain everyday cognitive functioning and improve quality of life in both cognitively healthy individuals and those with mild cognitive impairment or early dementia. The timing of intervention is important since delayed treatment may reduce the benefits of rehabilitation due to long-standing auditory deprivation and associated central changes [59]. This highlights the importance of early diagnosis and timely intervention, ideally before significant auditory–cognitive decline occurs.

From a public health perspective, hearing loss is a highly prevalent and potentially modifiable condition. Increasing awareness, improving access to hearing care, and reducing barriers and stigma of hearing aid use remain key priorities. Socioeconomic disparities also play an important role, as individuals from more deprived backgrounds are both more likely to experience hearing loss and less likely to receive adequate treatment [60]. Recent epidemiologic reviews emphasize that hearing aid use still falls behind hearing loss prevalence, in part because of affordability and accessibility factors [61]. Addressing these inequalities may therefore have broader implications for cognitive health at the population level.

## 7. Future Research Directions

Future research should address several unresolved questions regarding the relationship between hearing loss and dementia. From a preclinical perspective, studies are needed to better characterize the biological mechanisms linking auditory dysfunction and neurodegeneration, including the roles of neuroinflammation, vascular pathology, synaptic plasticity, and central auditory processing. The development of biomarkers capable of distinguishing hearing loss as a risk factor from hearing loss as an early manifestation of neurodegenerative disease represents a particularly important objective.

From a clinical perspective, future longitudinal studies should incorporate detailed audiological phenotyping, including speech-in-noise testing and measures of central auditory function, rather than relying solely on pure-tone audiometry. Additional randomized controlled trials are needed to determine whether hearing rehabilitation can modify long-term cognitive trajectories, identify the patient populations most likely to benefit, and establish the optimal timing of intervention. Integration of audiological, neuroimaging, cognitive, and biomarker data may provide a more comprehensive understanding of the auditory–cognitive relationship throughout aging.

## 8. Conclusions

Current evidence increasingly supports a multidimensional model in which hearing loss may function as a modifiable risk factor, a consequence of shared pathological processes, and an early clinical marker of neurodegeneration. Rather than supporting a single explanatory framework, available data suggest that causal, early-marker, and shared-pathology mechanisms may coexist and contribute to varying degrees across individuals and disease stages.

Although auditory rehabilitation cannot currently be considered a proven strategy for dementia prevention, it remains clinically important because it improves communication, reduces listening effort, and may support everyday cognitive functioning, particularly in individuals at increased risk. Given the high prevalence and potential modifiability of hearing loss, early identification and timely intervention should be integrated into routine clinical care for older adults.

Future research should focus on clarifying the long-term cognitive effects of hearing rehabilitation, identifying patient populations most likely to benefit, and defining optimal timing for intervention. More broadly, emerging evidence supports a paradigm shift in which hearing is increasingly viewed as a brain-based cognitive process rather than a purely sensory function. Within this framework, auditory dysfunction may represent a clinically accessible and potentially time-sensitive window into neurodegenerative disease, with important implications for precision aging medicine and future dementia prevention strategies.

## Figures and Tables

**Table 1:** Major epidemiological studies evaluating the association between hearing loss and cognitive decline or dementia.

Study	Design	Population/Sample	Hearing Assessment	Cognitive Outcome	Main Findings
Lin et al., 2011 [2]	Prospective cohort	Baltimore Longitudinal Study of Aging (*n* = 639)	Pure-tone audiometry	Incident dementia	Dementia risk increased with hearing loss severity; approximately fivefold higher risk in severe hearing loss
Lin et al., 2013 [17]	Longitudinal cohort	Health ABC Study (*n* = 1984)	Pure-tone audiometry	Cognitive decline	Hearing loss associated with accelerated decline in cognitive performance
Deal et al., 2015 [5]	Prospective cohort (ARIC)	Older adults (*n* = 253)	Audiometry	Executive function and processing speed	Stronger association with executive function and processing speed than memory
Gallacher et al., 2012 [6]	Prospective cohort	Caerphilly Study (*n* = 1057)	Auditory thresholds and phonological processing measures	Incident dementia	Central auditory processing measures predicted dementia more strongly than hearing thresholds
Fritze et al., 2016 [18]	Longitudinal administrative database study	German health claims cohort (*n* = 14,602)	Clinical diagnosis of hearing impairment	Incident dementia	Hearing impairment associated with increased dementia incidence
Loughrey et al., 2018 [3]	Systematic review and meta-analysis	36 studies (>20,000 participants)	Various methods	Cognitive impairment and dementia	Hearing loss associated with increased risk of cognitive impairment and dementia
Marinelli et al., 2022 [19]	Prospective population-based cohort (MCSA)	Older adults (*n* = 1200)	Formal behavioral audiometry	Incident dementia	Confirmed association between hearing loss and dementia using rigorous audiometric assessment

ARIC—Atherosclerosis Risk in Communities Study; Health ABC—Health, Aging and Body Composition Study; MCSA—Mayo Clinic Study of Aging.

**Table 2:** Competing interpretative models of the relationship between hearing loss and dementia: supporting evidence, limitations, and clinical implications.

Model	Supporting Evidence	Limitations	Clinical and Research Implications
Causal model	Hearing loss precedes cognitive decline; dose–response relationship; biologically plausible mechanisms	Residual confounding; lack of definitive long-term RCT evidence	Supports early hearing intervention and risk reduction strategies
Early marker model	Central auditory dysfunction may precede cognitive symptoms; auditory brain concept; neuroimaging evidence	Difficult to distinguish prodromal neurodegeneration from causal effects	Supports hearing assessment as a potential screening and risk stratification tool
Shared pathology model	Common vascular, metabolic, inflammatory, and aging-related pathways	Does not fully explain dose–response associations or intervention effects	Supports multidomain prevention approaches targeting shared risk factors

## Data Availability

No new data were generated or analyzed in this study. All information is based on previously published data, which are cited within the manuscript.

## Abbreviations

The following abbreviations are used in this manuscript:

ARIC	Atherosclerosis Risk in Communities
dB	Decibel
kHz	Kilohertz
